# HOW GERONTOLOGY PROGRAMS ARE MEETING CHALLENGES IN HIGHER EDUCATION

**DOI:** 10.1093/geroni/igad104.0634

**Published:** 2023-12-21

**Authors:** Mary Ann Erickson

## Abstract

Population aging means that gerontology education is increasingly relevant for all students, yet trends in higher education create existential challenges for many gerontology programs, who may not attract robust number of applicants or majors. In this symposium, educators from five different gerontology programs will share their programs’ particular challenges as well as a wide variety of strategies they have used to meet these challenges. The first presentation will show how recent losses at Ithaca College, including the Aging Studies major and independent department status, also come with opportunities for collaboration with health science and public health programs. The second presentation will describe the University of North Carolina at Wilmington’s approach of combining undergraduate and graduate programs to attract and retain students after being placed on a “low-productivity list” in the not-too-distant past. The third presentation will share how a new Occupational Endorsement Certificate at the University of Alaska Anchorage took the place of the deactivated gerontology minor. Colleagues from the University of Massachusetts Boston will describe their efforts to enhance diversity and grow program enrollment across undergraduate and graduate programs. Finally, the fifth presentation will describe how increased demand for general education courses as well as funding dependent on undergraduate major enrollment created challenges at the University of South Florida. This is a Directors of Aging Centers Interest Group Sponsored Symposium.

